# “The number of clients is increasing but the supplies are reducing”: provider strategies for responding to chronic antiretroviral (ARV) medicines stock-outs in resource-limited settings: a qualitative study from Uganda

**DOI:** 10.1186/s12913-019-4137-7

**Published:** 2019-05-15

**Authors:** Henry Zakumumpa, Flavia Matovu Kiweewa, Felix Khuluza, Freddy Eric Kitutu

**Affiliations:** 1 Makerere University, School of Public Health, Kampala, Uganda; 2 Makerere University Johns Hopkins Research Collaboration, Kampala, Uganda; 3 Pharmacy Department, University of Malawi, Blantyre, Malawi; 4 Pharmacy Department, Makerere University, Kampala, Uganda

**Keywords:** Stock-outs, ART, Health systems, Health services, Resource-limited settings, Supply chain management

## Abstract

**Background:**

Despite the increasing frequency of ARV medicines stock-outs in Sub-Saharan Africa, there is little research inquiring into the mitigation strategies devised by frontline health facilities. Many previous studies have focused on ‘upstream’ or national-level drivers of ARVs stock-outs with less empirical attention devoted ‘down-stream’ or at the facility-level. The objective of this study was to examine the strategies devised by health facilities in Uganda to respond to the chronic stock-outs of ARVs.

**Methods:**

This was a qualitative research design nested within a larger mixed-methods study. We purposively selected 16 health facilities from across Uganda (to achieve diversity with regard to; level of care (primary/ tertiary), setting (rural/urban) and geographic sub-region (northern/ central/western). We conducted 76 Semi-structured interviews with ART clinic managers, clinicians and pharmacists in the selected health facilities supplemented by on-site observations and documentary reviews. Data were analyzed by coding and thematic analyses.

**Results:**

Participants reported that facility-level contributors to stock-outs include untimely orders of drugs from suppliers and inaccurate quantification of ARV medicine needs due to a paucity of ART program data. Internal stock management solutions for mitigating stock-outs which emerged include the substitution of ARV medicines which were out of stock, overstocking selected medicines and the use of recently expired drugs. The external solutions for mitigating stock-outs which were identified include ‘borrowing’ of ARVs from peer-providers, re-distributing stock across regions and upward referrals of patients. Systemic drivers of stock-outs were identified. These include the supply of drugs with a short shelf life, oversupply and undersupply of ARV medicines and migration pressures on the available ARVs stock at case-study facilities.

**Conclusion:**

Health facilities devised internal stock management strategies and relied on peer-provider networks for ARV medicines during stock-out events. Our study underscores the importance of devising interventions aimed at improving Uganda’s medicines supply chain systems in the quest to reduce the frequency of ARV medicines stock-outs at the front-line level of service delivery. Further research is recommended on the effect of substituting ARV medicines on patient outcomes.

**Electronic supplementary material:**

The online version of this article (10.1186/s12913-019-4137-7) contains supplementary material, which is available to authorized users.

## Background

Over the past decade, universal access to antiretroviral therapy (ART) has been gathering momentum as a global health priority [1, 2]. In 2014, The Joint United Nations program on HIV/ AIDS (UNAIDS) unveiled the 90–90-90 targets which call for enrolling 90% of those diagnosed with HIV on sustained ART as well as achieving viral suppression in 90% of those enrolled on anti-retroviral therapy (ART) [3]. In 2015, the World Health Organization (WHO) released treatment guidelines recommending that all diagnosed as HIV positive be initiated in care irrespective of disease stage [4]. In 2017, the universal ‘test and treat’ (UTT) policy was implemented in several low and middle-income countries [5].

The attainment of these global health goals depends substantially on the capacity of health-systems to ensure sufficient stocks of antiretroviral drugs (ARVs) to meet the escalating demand for treatment especially at the front-line level of service delivery [6, 7].

The stock-outs of antiretroviral (ARV) medicines and related supply chain management bottlenecks are common in Sub-Saharan Africa [8–10]. To achieve viral suppression, patients on ART require an uninterrupted supply of ARVs. Interruptions in supplies of ARVs have been associated with HIV drug resistance and treatment failure as well as mortality in patients enrolled in care [11–13]. Hence, research that documents locally-derived mitigation strategies for responding to chronic antiretroviral drugs stock-outs, especially in resource-limited settings, is critical [14, 15].

Although there have been several global strategies and policy guidelines for responding to the frequent stock-out of antiretroviral medicines in resource-limited settings [16–18] and at the macro-level [6, 12, 19], there is a dearth of evidence at the front-line level of ART-providing organizations or an understanding of the strategies adopted at the meso or facility-level [20, 21] in response to the frequent stock-outs of antiretroviral medicines in Sub-Saharan Africa. A notable exception is a study by Mori and colleagues [8] in Tanzania. This study however more specifically focused on strategies by providers of avoiding changes in ART regimens in one district of Tanzania. To date, there is a paucity of data on strategies adopted at the frontline level of service delivery to mitigate the chronic stock-outs of antiretroviral medicines in Uganda and other countries with a high HIV burden. Previous studies have focused on the effect of stock-outs of ARV medicines on patient outcomes [11–13, 22].

### ART scale-up in Uganda and ARVs supply management system

Uganda piloted a national ART roll-out program between 2004 and 2009 with external donor support from The President’s Emergency Plan for AIDS relief (PEPFAR) and The Global Fund to fight AIDS, Malaria and Tuberculosis [23, 24]. The national ART roll-out program commenced initially at the level of national and regional referral hospitals and was subsequently extended to lower-level health facilities [25], including in small for-profit clinics [2]. Despite the availability of donor support for procurement of ARVs, especially from The Global Fund which funds 62% of HIV commodities in public facilities in Uganda [26], the stock-out of antiretroviral drugs at point-of-care is common. In Uganda, it is estimated that only 27% of district hospitals and 40% of lower-level health facilities are supplied with the quantities of essential medicines they request [6]. A 2016 performance audit by the Global Fund found that the stock-outs of essential medicines was pervasive in Uganda [27]. According to this report, the causes of stock-outs in Uganda include a critical shortage of personnel specialized in medicines supply chain management, drug theft at multiple levels, misappropriation of funds for medicines procurement, weak storage capacity at the central medical stores leading to expired drugs [27]. Indeed, studies have documented the insufficient and irregular supply of antiretroviral medicines in Uganda [6, 27, 28]. However, the strategies adopted at the level of providers in response to the chronic stock-outs of ARVs and to promote the sustainability of ART provision is an area that is under-explored. As is common with many Sub-Saharan African countries [8, 10–12], the supply chain management of antiretroviral drugs (ARVs) is especially weak in Uganda [6]. As the number of patients enrolled on ART continually increases, with more ART clinics being accredited, and the variety of ARVs becomes more diverse, the challenges of ARV stock-outs are compounded [19]. Early indications suggest that Uganda’s implementation of universal ‘test and treat’ which commenced in 2017 has exacerbated the challenge of stock-outs of anti-retroviral medicines due to a dramatic increase in new ART enrollments [29, 30].

The distribution of antiretroviral medicines in Uganda follows a dual-track supply chain mechanism based on ownership-type of a health facility. National Medical Stores (NMS) distributes ARVs to all public health facilities in the country which constitute over two-thirds of all providers of ART in Uganda. Commodities in public facilities are primarily funded by the Government of Uganda and the Global Fund for Fight AIDS, malaria and Tuberculosis. On the other hand, the private sector which incorporates Private- Not-for-Profit (PNFP) and Private-for-Profit (PFP) health facilities is supplied through the Joint Medical Stores (JMS) and Medical Access Uganda (MAUL). These drugs are in turn funded by the U.S. government through PEPFAR. The private sector accounts for about 20–30% of ART provision in Uganda [6, 26]. Public health facilities from the level of health centre IVs (sub-district) and higher-level hospitals [31] make formal requisitions for supply of medicines and other commodities based on patient data such as the number of patients and type of regimen (whether first or second line) and therefore the medicines they need. Hence, facility-level dynamics are influential on the adequacy of the stock of ARVs available for dispensing to patients. The objective of this study was to understand the strategies adopted at the level of health facilities in Uganda to respond to the chronic stock-out of antiretroviral medicines.

The findings reported here are derived from a larger study [23, 26, 32, 33] interrogating the sustainability of ART scale-up implementation in health facilities in Uganda from the perspective of WHO’s building blocks of the health system framework [34].

## Methods

### Research design

We aimed to understand the strategies devised to respond to the chronic stock-outs of antiretroviral medicines from the perspective of providers and to gain a sense of their operational contexts. To this end, a qualitative research design utilizing a multiple case-study approach was adopted [35, 36]. Semi-structured interviews were conducted with 78 health workers from 16 case-study facilities [37]. As shown in Table 1 [38]**,** on-site visits and observation approaches augmented interviewee data. Data were collected between April and June 2016.

**Table 1:** Processes for ensuring rigour in case-study analysis adapted from Gilson et al. (2012) [39]

PRINCIPLE	
Prolonged engagement	Multiple on-site visits were made to the case-study facilities across three weeks. Informal discussions were held with ART clinic managers, clinicians and pharmacists coupled with face-to-face interviews with multiple informants.
Use of theory	This study is derived from a larger mixed-methods study which is informed by the health systems dynamics frameworks by van Olmen et al. (2012) [33] which builds on the WHO’s building blocks of the health system framework (2007).
Case selection	Sixteen health facilities were purposefully selected from a nationally-representative sample of 195 health facilities across Uganda which participated in Uganda’s national emergency ART roll-out.
Sampling	We aimed for a sample that had appropriate representation of health facility demographics in Uganda with respect to a) setting (rural/urban), b) ownership-type (public, for-profit, not-for-profit)c) Level of care(tertiary, secondary, primary).
Multiple methods	Multiple methods were used including face-to-face interviews, document review and informal engagements with clinicians and the head of the HIV Clinic.
Triangulation	Case descriptions were constructed based on triangulation across multiple data sources (Questionnaire data, interviewee data and document review).
Peer debriefing and support	Data analysis involved a team-based process involving at least three authors at each of the stages.
Respondent validation	A data validation workshop was conducted with involving the head of the HIV clinic in 14 of the Participating health facilities.

### Study sites and selection

This qualitative study was nested within a larger two-phased mixed-methods sequential explanatory research design which is described elsewhere [23, 26, 32, 33]. Health facilities were selected in a two-stage process. In the first stage, 195 (out of 394) health facilities which participated in Uganda’s pilot national ART scale-up phase between 2004 and 2009 were selected [6]. In the second study phase, a multiple case-study [39] approach was adopted. To this end, 16 health facilities were purposively selected from those which participated in the first study phase [37] to achieve diversity with regard to a) ownership-type (public/private) b) setting (rural/urban) c)level of care in the Ugandan health system [40] (tertiary/ secondary/primary) d) regional representation (at least two health facilities were selected from each of Uganda’s 8 geographic sub-regions as designated by the Uganda Bureau of Statistics (South West, East Central, Mid-East, Mid-West, Central 2, Mid North, North East and Kampala)) [37]. The demographic characteristics of the case-study facilities are shown in Table 2.

**Table 2:** Health facility demographic information

	Accronym	Ownership-type	Level of care in ugandan health system	Setting	Geographic sub-region	Annual art patient load (as at june 2015)
1	PUB-001	PUBLIC	Referral Hospital	Urban	South West	24,408
2	PUB-002	PUBLIC	Referral Hospital	Urban	Kampala	2408
3	PUB-003	PUBLIC	Referral Hospital	Urban	Central 2	6414
4	PUB-004	PUBLIC	District Hospital	Urban	East Central	598
5	PUB-005	PUBLIC	Health centre IV	Peri-urban	Mid-East	458
6	PUB-006	PUBLIC	Health centre IV	Rural	Mid-north	2034
7	PUB-007	PUBLIC	Health centre IV	Rural	East-Central	263
8	PUB-008	PUBLIC	Health centre IV	Peri-urban	Mid-west	298
9	PUB-009	PUBLIC	Health centre IV	Rural	North East	126
10	PNFP-001	NOT FOR PROFIT	Referral Hospital	Urban	Kampala	4337
11	PNFP-002	NOT FOR PROFIT	Referral Hospital	Urban	East central	1727
12	PNFP-003	NOT FOR PROFIT	Health Centre IV	Rural	Mid-East	647
13	PNFP-004	NOT FOR PROFIT	Health Centre IV	Peri-urban	South West	402
14	PFP-001	FOR-PROFIT	Health centre III	Urban	Mid-West	324
15	PFP-002	FOR-PROFIT	Health Centre II	Urban	Central 2	29
16	PFP-003	FOR-PROFIT	Health Centre II	Rural	Mid-North	46

### Data collection

A semi-structured interview guide (Additional file 1) was constructed for use in conducting 78 interviews (with the head of the ART clinic, facility in-charge and pharmacist (in the case of large hospitals e.g. PUB 001, PUB 002 PNFP-001, PNFP-002) as well as clinicians of 16 case-study facilities. Table 3 lists the category of informants interviewed for this study. The interview questions were opened-ended and broadly aimed at facilitating a facility-based understanding of the facilitators and barriers to the long-term sustainability of ART scale-up implementation in Uganda [2, 23, 26, 32, 37]. The WHO ‘building blocks’ of the health system framework [33, 34] was utilized as a guiding analytical framework. We sought to gain a sense of the operational context of case-study facilities, the ‘inner context’ and the ‘outer context’ [41] factors contributing to the stock-outs of ART medicines at the facility-level as well as the broader systemic influences on ART stock availability at case-study facilities. We aimed to explore the decision space available to facility-level managers with regard to strategies for mitigating ARVs stock-outs. The original interview guide was pilot-tested and revised based on feedback from 12 ART clinic managers of health facilities outside the study sample [42].

**Table 3:** Category of interviewees (*n =* 78)

Head of ART clinic	*n = 16*
Facility in-charge	*n = 16*
Clinicians	*n = 39*
Pharmacists (in the case of higher-level hospitals)	*n = 7*
Total	*78*

The interview guide started off with general questions about health facility demographics (e.g. range of HIV services offered, number of patients on ART). The next phase of the interview was then devoted to fielding open-ended questions to elicit information relating to the strategies devised to respond to chronic ARV stock-outs.

Face-to-face interviews were conducted by the first author who holds a PhD in health systems with an academic background in the social sciences and an expertise in qualitative research [2, 23, 26, 32, 37]. The first author was assisted by two research assistants with extensive experience in qualitative research and health services research. We sought interviewees with substantial ART program experience especially those who had been in service during the commencement of ART services at participating health facilities during the emergency phase of national ART roll-out [6]. The face-to-face interviews were conducted in English and on average lasted between 45 and 60 min. For respondents’ convenience, interviews were conducted on-site at the health facilities in the offices of interviewees within the health facility premises. To supplement interview data, the first author and the two Research Assistants each maintained a daily diary in which they recorded observations [35] during interviews with ART clinic managers as well as the general characteristics of the health facilities such as infrastructure and patient volumes. The documented observations were then formalized into research memos [35].

### Data analysis

Our qualitative data analysis approach was broadly informed by Miles & Huberman (1994) [35]. We analyzed data in four major stages; a) *Data familiarization*: As a first step, the audio-recorded interviews were transcribed verbatim into text transcripts by the first author and two Research Assistants experienced in qualitative data analysis. This was followed by multiple readings of the interview transcripts by three authors for purposes of data familiarization (HZ, FM, and FK2) b) *Generating a coding framework*: Three authors devised an initial coding scheme based on multiple readings of interview transcripts (HZ, FM, and FK1). The three authors then independently read and analyzed the transcripts based on the generated coding scheme (HZ, FM, and FK2). Additional codes were generated for data not captured by the initial coding scheme c) *Data abstraction into thematic clusters:* The emergent codes were then grouped under thematic categories in joint peer-debriefing sessions involving all authors (HZ, FM, FK1, FK2) *d*) *Interpretation and synthesis:* Overall interpretation of the study results involved a team-based process involving all four authors which resolved discrepancies in interpretation through consensus. As recommended by Gilson, L. et al. [42], a respondent data validation workshop was conducted with ART clinic managers from 8 (of the 16) participating health facilities to ensure accuracy in interpretation of interviewee data. Their feedback and in-put informed the final analyses.

## Results

### Facility-level contributors to stock-outs

#### Inaccurate ART medicines quantification

Across our interviews with ART clinic in-charges it emerged that the frequent stock-outs of ARVs were partly attributed to the continually-increasing HIV client loads. It was indicated in the interviews that the increasing patient volumes were not being properly documented or adequately planned for by health facilities. As a result, accurate ART commodity quantification was impeded which in turn impacted on the requisitions to the national medicines supplier.

*‘Stock-outs of ARVs are a huge problem here because of the huge number of patients that keeps swelling all the time at our ART clinic. So the consumption rate of drugs is very high’ [ART clinic manager, PUB-003].*

#### Untimely orders of drugs

ART clinic managers conceded that stock-outs of ARVs were as a result of factors internal to the health facility as well as drivers from the external environment. Placing timely orders for ARV medicines with NMS (National Medical Stores) was described as critical to ensuring adequate stocks and regular supply of ARVs. However, due to a variety of factors, including occasional surges in workloads, health workers reported that, at times, they did not submit these orders on time. Orders were said to be to be based on a bi-monthly (once every two months) order cycle.

*‘In fact one of the problems (of stock outs) is with us. One of the staff who worked here before being transferred was not very keen and did not place orders for ARVs on time s and that is what led to stock-outs. We cannot blame somebody else for that’ [Facility in-charge, PUB-004].*

### Internal stock management strategies

#### ARV medicines substitutions

A strategy commonly cited across participating health facilities was that of drug substitution for ARVs which were out of stock. Clinicians described having to substitute drugs for patients on ART if a specific class of drugs they were taking was out of stock. This was said to be a common occurrence during patient visits to ART clinics for routine reviews and drug re-fills. Interviewees described being frequently compelled to switch regimens for patients on ART due to shortages rather than out of clinical need.

*‘Drug stock outs of ARVs are chronic here. There is irregular supply of commodities and sometimes certain drug combinations are not in stock. The number of clients is increasing but the supplies are reducing. In such instances, we can substitute. If drug X is not in stock, we can prescribe an alternative. We play around with drugs when we don’t have certain combinations. And we counsel our patients to accept the alternative drugs’ [ART program manager, PNFP-001].*

In PUB-007, a sub-district health facility, it was reported that they often experienced prolonged stock-out of both ARVs and anti-Tuberculosis (TB) drugs lasting for as long as 3 months and longer and that during these periods, clinicians were compelled to prescribe ARVs that they had in stock but which were no longer recommended regimens following revision of the national ART treatment guidelines.

#### Overstocking select ARVs

Interviewees perceived the chronic stock outs of ARVs as emanating from an inefficient national commodities supply chain system and as such, stock outs were always anticipated. ART clinic managers indicated they deliberately overstocked ARVs to mitigate the frequent interruptions in supply.

In seven of the case-study facilities, overstocking was mentioned as a strategy for averting ARV medicines stock-outs which providers described as frequent.

*‘Our implementing partner had a mechanism of overstocking and alternative supply-lines approaches because these stock-outs are actually common.’ [Facility in-charge, PUB-001].*

Specific medicines were repeatedly mentioned as candidates for overstocking. These include co-trimoxazole tablets (septrin) which is prescribed in HIV-infected persons to reduce the risk of opportunistic infections, more especially diarrhea, malaria and pneumonia. Dapsone was also frequently mentioned as a drug which was overstocked to prevent interruption in supply. Dapsone is prescribed to people with HIV as preventive therapy against opportunistic infections (OIs) in those patients allergic to co-trimoxazole.

#### Utilizing recently expired drugs

The facility in-charge of PUB-007 indicated that during a recent prolonged stock-out of second-line ARVs lasting between 3 and 6 months, they were forced to prescribe drugs which had they had in store but had expired by about three months.

*‘When we experienced a prolonged stock-out last year, we got advice from our implementing partner that if there is a district in our region which has drugs that have just recently expired we can use them. For example, we are using second-line drugs that recently expired in September (three months back). We were told that those ones can serve but pharmaceutically we are not allowed to do that’ [ART clinic manager, PUB-007].*

#### In-house extra purchases of medicines

Private health facilities reported making additional purchases of ARVs from their own internal funds if specific drugs were not supplied by Uganda’s Joint Medical Stores (JMS) and Medical Access Uganda Limited (MAUL). In a private employee clinic run by a multi-national tea estate company based in Western Uganda, the parent company often made stand-in purchases of ARVs if there were delays in supplies by their principal supplier.

*‘We usually purchase ARVs that are not supplied by Medical Access (main supplier) using our own (company) funds to ensure that ART services continue without interruption’ [Facility in-charge, PFP-001].*

### Leveraging external networks to mitigate stock-outs

#### ‘Borrowing of drugs’ within peer-provider networks

Across our interviews with ART clinic managers, the sharing or ‘borrowing’ of drugs within peer-provider networks during stock-out events was a frequently reported strategy across health facility ownership-type. The borrowing of anti-retroviral medicines from peer providers was often with respect to specific combinations that were out of stock. Second-line ARVs stood out in this regard. ART clinic managers reported that more general stock-outs of HIV commodities were common. They described instances when the national medicines supplier (NMS) delayed to re-supply health facilities or in situations when a prolonged general stock-outs of ARVs was being experienced nationally or when specific geographic sub-regions of Uganda were being especially affected*.*

The borrowing of drugs among peer-providers in our sample of health facilities occurred in two principal networks namely; a) faith-based alliances and b) within neighborhood or regional networks of providers.

##### Faith-based networks

Within the faith-based networks, we found that health facilities under the umbrella of the Uganda Catholic Medical Bureau (UCMB) borrowed drugs from within their networks especially those at a similar level of care and those located in the same district. For instance, PNFP-001 borrowed drugs from a general hospital affiliated with UCMB in central Uganda.

*‘We do a lot of internal borrowing. We often experience drug stock outs. In such cases, we borrow drugs from sister hospitals such as (names of three catholic-founded providers) and we replace their drugs when we replenish our stock’ [ART program manager, PNFP-001].*

Our findings show that the borrowing of ARVs was common among health facilities in the same neighborhood or those in close physical proximity to each them. PUB-001, a regional referral hospital reported borrowing drugs from a neighboring large not-for-profit hospital in South-Western Uganda.

*‘What we do is look around other facilities which have some supplies. We borrow drugs from a neighboring NGO provider when we experience a stock-out or if we don’t have a particular combination in stock’ [ART clinic manager, PUB-001].*

Borrowing of drugs was reported across both public and private providers. Two private clinics (PFP-002, PFP-006) borrowed drugs from a neighbouring for-profit (PFP-003) clinic. A network of private clinics participating in the study which were owned by a multinational tea estate company that operated employee clinics within tea estates spread across Western Uganda reported sharing ARVs within their network of clinics.

*‘We can borrow drugs from our sister clinics if our HIV or TB drugs are not delivered on time (from supplier). There are clinics in our network of employee clinics run by the Tea Company such as (name of tea estate A) and (name of tea estate B). When they have excess septrin (co-trimoxazole) we can request and they give us’ [Facility in-charge, PFP-001].*

#### Stock re-distribution across regions

A PEPFAR implementing organization with responsibility for programmatic oversight of HIV services in the region of Eastern Uganda was reported to rationalize the stock of ARVs across all health facilities in this region. ART clinic in-charges indicated that the PEPFAR implementing organization often picked ARVs from health facilities with stock to spare and distributed to health facilities experiencing shortages. In this way, it was reported, that the stock of ARVs available in a geographic sub-region was redistributed and shared across all health facilities to prevent interruptions in supply but also to ensure stock management efficiencies across the geographic sub-region.

*‘Recently when we had a stock-out of second-line ARVs. We ran to neighbouring districts which still had some and they gave us. When we also have (stock) and they don’t have, we also give them’ [ART clinic manager, PUB-002]*

*‘We redistribute from other health facilities which have stock to those which don’t have so at least we keep moving on’ [Facility in-charge, PUB 006]*

#### Upward referrals

A related finding is that during stock-out events, providers often referred patients to higher-tier hospitals which were perceived to be better stocked with ARVs. Upward referrals were frequently made to enable patients access ARVs as opposed to the referrals being made on the basis of clinical criteria.

*‘During the two months when we had stock-outs of ARVs, patients were off treatment for two months. There was no immediate solution. So, you refer patients to the district hospital so that they can get drugs’ [ART clinic In-charge, PUB-004]*

### Systemic drivers of ARV stock-outs

#### Long lead times

Facility in-charges revealed that the lead times (the time-lag between placing orders for ARVs and their subsequent delivery) were long and lasted for as long as three months or even a year. Participants indicated they had grown accustomed to long lead times and that they actually expected them. Forecasting the individual needs of a health facility was reported as helpful in overcoming the challenge of bottlenecks in the supply chain which was described as common. At times, shortages in the supply chain were reported to be with respect to specific combinations.

*‘We have a bi-monthly order cycle. The challenge is that JMS sometimes doesn’t have a certain ARV. A certain commodity X or Y. Sometimes the shortage for certain combinations are more common and hence you plan accordingly’ [ART program Manager, PNFP-001].*

#### Close-to-expiry drugs

ART clinic in-charges across our case-study facilities reported that the ARVs delivered to health facilities often had a short shelf life. They complained that the commodities delivered to them often had 2–3 months left before expiry or even shorter and it that it was common for recently delivered drugs to expire in their stores.

*‘Sometimes they bring us drugs which are about to expire. They bring them when they have a short shelf life. And they tell us,’ continue using them’. You see that is the challenge’ [ART clinic manager, PUB-002].*

#### Maldistribution of drugs

Interviews with facility in-charges and ART clinic managers revealed that ‘erratic’ supply of ARVs was common. Examples were given of oversupply of specific ARVs at one health facility when the nearest health facilities (in the same neighbourhood) was experiencing a stock-outs of that specific drug. This scenario is described by an interviewee.

*‘You find that a health facility instead of getting 10 tins of septrin (co-trimoxazole) they actually get 20, because of one reason or another. But then a nearby health facility doesn’t have any. There are cases of erratic stock. So, we share. You find that a health facility here is suffering but another health facility in a neighboring district has extra stock. But we are in one regional network. We share information about supply and logistics with each other’ [Facility in-charge, PUB-002].*

#### Migration pressures on ARV stock

A representative from a district hospital participating in the study [PUB-003] indicated that the hospital serves a broader base of clients than those hailing from the district because it was located in an area of Uganda bordering two neighbouring countries. Some of the clients for the ART clinic were walk-in patients from two neighbouring countries due to porous borders but also because it hosts a large refugee community [43] which exacerbates commodities stock-out bottlenecks because of a substantial number of walk-in patients who were not on their books and who were not often not factored in the quantification of the needed ARVs.

*‘We behave like an international hospital. We even have clients coming in from (neighbouring) Rwanda and Congo (DRC). So, the consumption rate of commodities is high because of that’ [Nurse, PUB-005].*

Additionally, health workers pointed out increasing cases of patients crossing from neigbouring districts in Uganda seeking HIV care at PUB-003. In all, health workers mentioned five neigbouring districts from which patients hailed. They complained about serving huge client loads from neighbouring districts who they had not planned for in terms of commodities which put pressure on the stock of ARVs available to dispense to their regular and registered clients.

## Discussion

The stock-out of antiretroviral and concomitant drugs is an increasingly chronic bottleneck in HIV service delivery in Uganda and the broader Sub-Saharan Africa region [8, 10–12]. Previous approaches have examined this phenomenon from a macro-level lens such as studies interrogating national-level drivers of stock-outs in countries with weak medicines supply chain systems [10, 12, 17, 19, 31]. However, there has been limited empirical attention devoted to strategies devised at the front-line level of service delivery in response to the chronic stock-outs of antiretroviral medicines [44].We aimed to address this gap in this paper. We found that the ‘borrowing’ of ARVs from peer-providers was a common strategy adopted during stock-out events in Uganda. Drug substitution which entails selecting alternative ARVs for those that are out of stock was a frequently cited strategy. Providers reported that due to the frequent occurrence of stock-out events, overstocking of ARVs and concomitant drugs was adopted as a mitigation measure in anticipation of bottlenecks in the supply of ARVs. Overall, providers described the national medicines supply chain system in Uganda as inefficient as manifested in the irregular and insufficient supply of ARVs which was described as chronic. A USAID study [39] found that 73% of for-profit providers in Uganda identified ‘inadequate supply of ARVs’ as the most important constraint in ART service delivery.

### Facility-based contributors to ARV stock-outs

An important finding of this study is that providers perceived the phenomenon of the stock-out of antiretroviral drugs as one arising from a complex interaction between ‘inner setting’ and ‘outer setting’ factors [41]. Contrary to the dominant discourse on inefficient centralized medicines supply chain systems in Sub-Saharan Africa especially those involving national-level actors [10, 17, 19, 31] providers conceded that part of the problem of the chronic stock-outs originated at the facility-level due to a number of factors including inaccurate quantification of ARV needs and untimely orders for medicines with the National Medical Stores (NMS). The paucity of basic ART program records relating to the number of patients enrolled on ART was cited as one of the impediments to the accurate forecasting and requisitions of ARVs from the national medicines supplier. In suggesting a link between health information systems and medicines supply, we offer some empirical credence to previous studies calling for a ‘systems thinking’ perspective in understanding bottlenecks in accessing essential medicines [6, 7, 33]. Bhojoni and colleagues in a study on diabetes control in India found that access to diabetes medicines at the frontline level of service delivery was affected by a complex interaction in health system sub-components [45]. Bigdelli and colleagues [46] have highlighted the complex mix of factors impacting on access to medicines and the interconnections in health system sub-systems that influence medicines supply chains [6].

### External solutions for mitigating stock-outs

In this study, we found that ‘borrowing’ or sharing ARVs among peer-provider networks was common. Although the borrowing of drugs during stock-outs has been previously reported among providers in resource-limited settings [28], including a grey literature report which indicated that 50% of for-profit clinics in Uganda shared ARV medicines during stock-outs [47], the unique contribution of this study is in adding more characterization to this phenomenon. Our study identifies two principal networks with in which this ‘borrowing’ of drugs occurs namely; faith-based provider alliances as well as within loose affiliations of providers based in the same geographic sub-regions or neighbor-hood fraternities. We found that drug substitution of specific ART regimens which were out of stock was widespread in participating health facilities. A study by Mori and colleagues [8] in Tanzania found that providers frequently prescribed alternative regimens when specific combination of ARVs were out of stock. In this connection, studies have documented the phenomenon of ‘improvisation’ [11, 28] as a response to the stock-outs of essential medicines in countries in resource-constrained settings. Another study by Gils and colleagues [48] in Congo found that drug substitutions for ARVs were common in Congo with the attendant concerns of effects on patient outcomes and medical ethics. Contrary to the findings of a study conducted in Eastern Uganda by Windisch and colleagues [6] which found that shortages for essential drugs other than ARVs, were more apparent, we found that the stock-outs of ARV medicines was prominent indeed across participating health facilities at the time of data collection. This may be a reflection of the escalating HIV client loads since the previous study was conducted.

### Systemic impediments to the availability of sufficient stocks of ARVs

We found that cases of erratic stock involving under-supply and over-supply of specific drugs were common in participating health facilities [6, 49]. It was common to find that a health facility experienced a shortage of a specific ARV when a neighbouring facility had an excess supply of the same regimen. This scenario of ‘scarcity amidst plenty’ points to weaknesses in the current medicines supply chain system in Uganda which may contribute to ‘artificial’ shortages that can be ameliorated through evolving a more efficient and robust supply management system by Uganda’s National Medical Stores (NMS) and other national players. A recent PEPFAR report [48] notes that in Uganda ‘There are serious concerns regarding HIV commodity security because of systemic issues resulting from a generally weak supply chain, flawed forecasting and quantification, poor Uganda government procurement practices, and forward funding of commodities by The Global Fund’.

Our study illuminates the *systemic* constraints underpinning ARVs medicines availability at the frontline level of service delivery which poses challenges to meeting new global HIV epidemic control targets such as universal ‘test and treat’ [1] in Uganda and other similar resource-limited settings [29, 30] that calls for supply chain reforms and other remedial responses [50]. In its latest published annual national HIV program targets, PEPFAR, the predominant donor in Uganda [26] declares one of its principal goals as ‘ improving the quality of the logistics management information systems to ensure accurate and timely ordering and instituting systems that provide timely real time stock status data at health facilities so as to inform decision-making’ [48]. These reforms will be critical to the successful implementation of the topical Differentiated ART Service Delivery (DSD) [51, 52], which provides for multi-month dispensing [52] or an arrangement where clinically stable patients are provided with ARVs lasting as long as 3–6 months in the bid to decongest ART clinics and relieve pressure on over-burdened African health- systems [53].

### Limitations

This study utilized a case-study research design which is amenable to in-depth and contextualized understanding of phenomena [39]. We deliberately adopted a case-study design to contribute to an in-depth, facility-based understanding of the strategies adopted at the front-line level of service delivery in Uganda in response to the chronic stock-out of ARVs. Case-study designs have inherent limitations in statistical generalizability of findings which we wish to acknowledge here [39]. Our study was conducted from the perspective of providers. Additional interviews such as with national-level policymakers could have elicited a more broad-based picture. One of the strengths of this study was a sample selection of health facilities that aimed for diversity with regard to ownership-type(public/private), level of care in the Ugandan health system (tertiary/ primary) and diversity in the geographic sub-regions of Uganda.

## Conclusion

Health facilities devised pragmatic strategies for reducing interruptions in the supply of ARV medicines through devising internal stock management arrangements and relying on peer-provider networks during stock-out events. Our study underscores the importance of devising interventions aimed at improving Uganda’s medicines supply chain system in the quest to reduce the frequency of ARVs stock-outs at the front-line level of service delivery. Further research is recommended on the effect of ARVs drug substitution on patient safety and outcomes.

## Additional file

Additional file 1: Semi-structured interview guide. Word text of semi-structured interview guide. (DOCX 13 kb)

## Abbreviations

AIDS: Acquired Immune Deficiency Syndrome
ART: Antiretroviral therapy
ARV: Antiretroviral
ARVs: Anti-retrovirals
GFATM: Global Fund to Fight HIV/AIDS, Tuberculosis and Malaria
HIV: Human Immunodeficiency Virus
JMS: Joint Medical Store
MOH: Ministry of Health
NMS: National Medical Stores
OIs: Opportunistic infections
PEPFAR: United States President’s Emergency Plan for AIDS Relief
SSA: Sub-Saharan Africa
UNAIDS: The Joint United Nations Program on HIV/AIDS

## References

[1] Bekker LG, Alleyne G, Baral S, Cepeda J, Daskalakis D, Dowdy D, Dybul M, Eholie S, Esom K, Garnett G, Grimsrud A (2018). Advancing global health and strengthening the HIV response in the era of the sustainable development goals: the international AIDS society—lancet commission. Lancet.

[2] Zakumumpa H, Bennett S, Ssengooba F (2016). Accounting for variations in ART program sustainability outcomes in health facilities in Uganda: a comparative case study analysis. BMC Health Serv Res.

[3] Oberth G, Whiteside A (2016). What does sustainability mean in the HIV and AIDS response?. Afr J AIDS Res.

[4] Bemelmans M, Baert S, Negussie E, Bygrave H, Biot M, Jamet C, Ellman T, Banda A, van den Akker T, Ford N (2016). Sustaining the future of HIV counselling to reach 90-90-90: a regional country analysis. J Int AIDS Soc.

[5] Camlin CS, Seeley J, Viljoen L, Vernooij E, Simwinga M, Reynolds L, Reis R, Plank R, Orne-Gliemann J, McGrath N, Larmarange J (2016). Strengthening universal HIV ‘test-and-treat’approaches with social science research. AIDS (London, England).

[6] Windisch R, Waiswa P, Neuhann F, Scheibe F, de Savigny D (2011). Scaling up antiretroviral therapy in Uganda: using supply chain management to appraise health systems strengthening. Glob Health.

[7] Schneider H, Blaauw D, Gilson L, Chabikuli N, Goudge J (2006). Health systems and access to antiretroviral drugs for HIV in southern Africa: service delivery and human resources challenges. Reproductive health matters.

[8] Mori AT, Owenya J (2014). Stock-outs of antiretroviral drugs and coping strategies used to prevent changes in treatment regimens in Kinondoni District, Tanzania: a cross-sectional study. J Pharm Policy Pract.

[9] David PM (2014). Towards the embodiment of biosocial resistance? How to account for the unexpected effects of antiretroviral scale-up in the Central African Republic. Glob Public Health.

[10] Poku RA, Owusu AY, Mullen PD, Markham C, McCurdy SA (2017). HIV antiretroviral medication stock-outs in Ghana: contributors and consequences. Afr J AIDS Res.

[11] Pasquet A, Messou E, Gabillard D, Minga A, Depoulosky A, Deuffic-Burban S, Losina E, Freedberg KA, Danel C, Anglaret X, Yazdanpanah Y (2010). Impact of drug stock-outs on death and retention to care among HIV-infected patients on combination antiretroviral therapy in Abidjan, Côte d'Ivoire. PLoS One.

[12] Harries AD, Schouten EJ, Makombe SD, Libamba E, Neufville HN, Some E, Kadewere G, Lungu D (2007). Ensuring uninterrupted supplies of antiretroviral drugs in resource-poor settings: an example from Malawi. Bull World Health Organ.

[13] Kranzer K, Ford N (2011). Unstructured treatment interruption of antiretroviral therapy in clinical practice: a systematic review. Tropical Med Int Health.

[14] Piot P, Karim SS, Hecht R, Legido-Quigley H, Buse K, Stover J, Resch S, Ryckman T, Møgedal S, Dybul M, Goosby E (2015). Defeating AIDS—advancing global health. Lancet.

[15] Soni A, Gupta R (2009). Bridging the resource gap: improving value for money in HIV/AIDS treatment. Health Aff.

[16] Gilks CF, Crowley S, Ekpini R, Gove S, Perriens J, Souteyrand Y, Sutherland D, Vitoria M, Guerma T, De Cock K (2006). The WHO public-health approach to antiretroviral treatment against HIV in resource-limited settings. Lancet.

[17] Larson C, Burn R, Minnick-Sakal A, Douglas MO, Kuritsky J (2014). Strategies to reduce risks in ARV supply chains in the developing world. Glob Health Sci Pract.

[18] World Health Organization. Antiretroviral Procurement and supply chain management. 2014. Retrieved 29 Oct 2018 from: http://apps.who.int/medicinedocs/en/d/Js21613en/

[19] Schouten EJ, Jahn A, Ben-Smith A, Makombe SD, Harries AD, Aboagye-Nyame F, Chimbwandira F (2011). Antiretroviral drug supply challenges in the era of scaling up ART in Malawi. J Int AIDS Soc.

[20] Gilson L (2012). Health Policy and Systems Research A methodology reader.

[21] Otiso L, McCollum R, Mireku M, Karuga R, de Koning K, Taegtmeyer M (2017). Decentralising and integrating HIV services in community-based health systems: a qualitative study of perceptions at macro, meso and micro levels of the health system. BMJ Global Health.

[22] Brennan AT, Bor J, Davies MA, Conradie F, Maskew M, Long L, Sanne I, Fox MP (2017). Tenofovir stock shortages have limited impact on clinic-and patient-level HIV treatment outcomes in public sector clinics in South Africa. Tropical Med Int Health.

[23] Zakumumpa H, Bennett S, Ssengooba F (2017). Modifications to ART service delivery models by health facilities in Uganda in promotion of intervention sustainability: a mixed methods study. Implement Sci.

[24] Zakumumpa H, Kwiringira J, Rujumba J, Ssengooba F (2018). Assessing the level of institutionalization of donor-funded anti-retroviral therapy (ART) programs in health facilities in Uganda: implications for program sustainability. Glob Health Action.

[25] Chan AK, Ford D, Namata H, Muzambi M, Nkhata MJ, Abongomera G, Mambule I, South A, Revill P, Grundy C, Mabugu T (2014). The Lablite project: a cross-sectional mapping survey of decentralized HIV service provision in Malawi, Uganda and Zimbabwe. BMC Health Serv Res.

[26] Zakumumpa H, Bennett S, Ssengooba F (2017). Alternative financing mechanisms for ART programs in health facilities in Uganda: a mixed-methods approach. BMC Health Serv Res.

[27] The Global Fund. Audit of Global Fund grants in Uganda. Office of the Inspector General. Retrieved 5 Apr 2019 from: https://www.theglobalfund.org/en/oig/updates/2016-02-26-audit-of-global-fund-grants-in-uganda/. 2016.

[28] Muyinda H, Mugisha J (2015). Stock-outs, uncertainty and improvisation in access to healthcare in war-torn northern Uganda. Soc Sci Med.

[29] Zakumumpa H. Is Uganda ready for ‘test and treat’ in HIV fight? The Observer, Kampala, Uganda. Retrieved 30 Jan 2019 from: https://observer.ug/viewpoint/56553-is-uganda-ready-for-test-and-treat-in-hiv-fight.html

[30] Nelson G. Tested but untreated: how Uganda’s new AIDS efforts are leading to drug shortages. PRI. Retrieved 29 Oct 2018 from: https://www.pri.org/stories/2018-08-07/tested-untreated-how-ugandas-new-aids-efforts-are-leading-drug-shortage.

[31] Trap B, Musoke R, Kirunda A, Oteba MO, Embrey M, Ross-Degnan D (2018). Article 2: longitudinal study assessing the one-year effects of supervision performance assessment and recognition strategy (SPARS) to improve medicines management in Uganda health facilities. J Pharm Policy Pract.

[32] Zakumumpa H, Taiwo MO, Muganzi A, Ssengooba F (2016). Human resources for health strategies adopted by providers in resource-limited settings to sustain long-term delivery of ART: a mixed-methods study from Uganda. Hum Resour Health.

[33] Zakumumpa H, Dube N, Damian RS, Rutebemberwa E (2018). Understanding the dynamic interactions driving the sustainability of ART scale-up implementation in Uganda. Glob Health Res Policy.

[34] Mounier-Jack S, Griffiths UK, Closser S, Burchett H, Marchal B (2014). Measuring the health systems impact of disease control programmes: a critical reflection on the WHO building blocks framework. BMC Public Health.

[35] Miles MB, Huberman AM, Huberman MA, Huberman M. Qualitative data analysis: An expanded sourcebook: Sage; 1994. https://uk.sagepub.com/en-gb/afr/qualitative-data-analysis/book239534

[36] Tong A, Sainbury P, Craig J (2007). Consolidated criteria for reporting qualitative research (COREQ): a 32-item checklist for interviews and focus groups. Int J Qual Health Care.

[37] Zakumumpa H, Rujumba J, Kwiringira J, Kiplagat J, Namulema E, Muganzi A (2018). Understanding the persistence of vertical (stand-alone) HIV clinics in the health system in Uganda: a qualitative synthesis of patient and provider perspectives. BMC Health Serv Res.

[38] Gilson L, Hanson K, Sheikh K, Agyepong IA, Ssengooba F, Bennett S (2011). Building the field of health policy and systems research: social science matters. PLoS Med.

[39] Yin RK. Case study research design and methods third edition. Applied social research methods series. 2003;5.

[40] Ministry of Health, Health Systems 20/20, and Makerere University School of Public Health. April 2012. Uganda Health System Assessment 2011. Kampala, Uganda and Bethesda, MD: Health Systems 20/20 project, Abt Associates Inc. Retrieved 14 Jan 2019 from: https://www.hfgproject.org/uganda-health-system-assessment-2011/

[41] Damschroder LJ, Aron DC, Keith RE, Kirsh SR, Alexander JA, Lowery JC (2009). Fostering implementation of health services research findings into practice: a consolidated framework for advancing implementation science. Implement Sci.

[42] Kyayise A, Kyeyagalire R, Livesley N, Kirunda I, Tumwesigye B. Private-for profit HIV/AIDS care in Uganda: an assessment. 2008. Retrieved 5 Mar 2019 from: https://www.urc-chs.com/sites/default/files/UgandaPFPassessmentfulltechnicalreport_USltr.pdf.

[43] PEPFAR. Uganda Country Operational Plan (COP) 2018: Strategic direction summary. Retrieved 29 Jan 2019 from: https://www.google.com/url?sa=t&rct=j&q=&esrc=s&source=web&cd=1&ved=2ahUKEwiyofz6oZPgAhXzsHEKHVwbDA4QFjAAegQICRAB&url=http%3A%2F%2Fwww.pepfar.gov%2Fdocuments%2Forganization%2F285851.pdf&usg=AOvVaw3VqVjCOwAV6SUXphpSSBl_.

[44] Berhanemeskel E, Beedemariam G, Fenta TGHIV (2016). AIDS related commodities supply chain management in public health facilities of Addis Ababa, Ethiopia: a cross-sectional survey. J Pharm Policy Pract.

[45] Bhojani U, Devedasan N, Mishra A, De Henauw S, Kolsteren P, Criel B (2014). Health system challenges in organizing quality diabetes care for urban poor in South India. PLoS One.

[46] Bigdeli M, Jacobs B, Tomson G, Laing R, Ghaffar A, Dujardin B, Van Damme W (2012). Access to medicines from a health system perspective. Health Policy Plan.

[47] Meloni ST, Chaplin B, Idoko J, Agbaji O, Akanmu S, Imade G, Okonkwo P, Murphy RL, Kanki PJ (2017). Drug resistance patterns following pharmacy stock shortage in Nigerian antiretroviral treatment program. AIDS Res Ther.

[48] Gils T, Bossard C, Verdonck K, Owiti P, Casteels I, Mashako M, Van Cutsem G, Ellman T (2018). Stockouts of HIV commodities in public health facilities in Kinshasa: barriers to end HIV. PLoS One.

[49] USAID. The health initiatives for the private sector (HIPS) project. Final evaluation report, January 2013. http://pdf.usaid.gov/pdf_docs/Pdacu928.pdf.

[50] DiCicco-Bloom B, Crabtree BF (2006). The qualitative research interview. Med Educ.

[51] Duncombe C, Rosenblum S, Hellmann N, Holmes C, Wilkinson L, Biot M, Bygrave H, Hoos D, Garnett G (2015). Reframing HIV care: putting people at the Centre of antiretroviral delivery. Tropical Med Int Health.

[52] Prust ML, Banda CK, Nyirenda R, Chimbwandira F, Kalua T, Jahn A, Eliya M, Callahan K, Ehrenkranz P, Prescott MR, McCarthy EA (2017). Multi-month prescriptions, fast-track refills, and community ART groups: results from a process evaluation in Malawi on using differentiated models of care to achieve national HIV treatment goals. J Int AIDS Soc.

[53] Barker C, Dutta A, Klein K (2017). Can differentiated care models solve the crisis in HIV treatment financing? Analysis of prospects for 38 countries in sub-Saharan Africa. J Int AIDS Soc.

