# Supplementary material for: “The number of clients is increasing but the supplies are reducing”: provider strategies for responding to chronic antiretroviral (ARV) medicines stock-outs in resource-limited settings: a qualitative study from Uganda
Source: BMC Health Serv Res. 2019 May 15;19:312. doi: 10.1186/s12913-019-4137-7 (PMC6521347; doi:10.1186/s12913-019-4137-7)
Supplement: Supplementary file 1 — Semi-structured interview guide. Word text of semi-structured interview guide. (DOCX 13 kb) [file 12913_2019_4137_MOESM1_ESM.docx]

**INTERVIEW TOPIC GUIDE**

*Introductory questions*

**Health facility demographics**

Location (Rural/ Urban)

Level of care (Primary/ Secondary/ Tertiary)

Ownership-type (Public/ Private for-profit/ Private not-for-profit)

Range of HIV services offered (ART, HIV testing/ PMTC (Prevention of mother to child transmission)

HIV client loads (High volume/ Low volume site)

*Open questions*

***Facility-level dynamics and stock-outs***

What factors contribute to stock-outs of antiretroviral medicines at the facility-level?

- *Prompts: planning, utilization of stock, orders for drugs, ‘inner context factors, views and experiences*

***Internal stock mitigation strategies***

What mitigation strategies do you devise internally as a health facility when you experience stock outs of antiretroviral medicines?

How does your health facility prepare for stock outs of antiretroviral medicines, if at all?

- *Prompts: including how does your health facility prevent stock-outs, views and experiences*

***External solutions for mitigating stock-outs***

What strategies does your health facility adopt to mitigate stock-outs from the external environment?

- *Prompts: potential sources from outside the health facility, past experience*

***Systemic influences on ARV stock availability***

What factors contribute to the stock-out of antiretroviral medicines that appear to be out of the health facility’s control?

- *Prompts: Broader health-system factors, suppliers, HIV clients, demand for ARVs, previous experience*
- **Note: Additional follow-up questions were asked, as appropriate, with each participant.*
